# Exploring mental health stigma among chinese-english bilinguals: Dual-process model of emotional competence, flipped learning readiness, and academic performance in Mainland Chinese Universities

**DOI:** 10.3389/fpsyt.2022.1001796

**Published:** 2022-09-12

**Authors:** Lihua Pei

**Affiliations:** Faulty of International Studies, Henan Normal University, Xinxiang, China

**Keywords:** mental health stigma, flipped learning readiness, emotional competence, academic performance, Chinese-English bilinguals, dual-process model

## Abstract

Due to severe global competition and performance-related academic challenges, Chinese students are compelled to learn English and become bilinguals despite their non-English majors. Consequently, these students frequently experience psychological issues, including mental health stigma. Hence, the present study aims to explore the psychological factors associated with the academic performance of Chinese-English bilinguals as an outcome of their emotional competence, flipped learning readiness, and mental health stigma. Drawing on data from 448 Chinese-English bilingual students in universities in mainland China, the results based on structural equation modeling (SEM) indicated that their academic performance, flipped learning readiness, and emotional competence are negatively influenced by their mental health stigma. Moreover, the findings also validate that both flipped learning readiness and emotional competence significantly mediate the indirect effects of mental health stigma on the academic performance of bilinguals. The study's implications offer new and compelling evidence on the primary issue of mental health stigma among Gen Z bilingual students to raise deterrence against this psychological menace through collaboration across policymakers, academics, and mental healthcare providers.

## Introduction

Globally, individuals who speak multiple languages gain manifold advantages in various domains of work (e.g., inspiring creativity and productivity as highly valued employees) and accelerate long-lasting relationships in social life. Nevertheless, bilingualism also translates into severe psychological issues (e.g., mental health stigma) that detract individuals from seeking optimal performance (1). More and more Chinese students are learning the global lingua franca “English” as a primary subject. Even non-English major track students in China (e.g., medicine, engineering, and computer science) are reading their major-related books in English (2). Increasing performance-related requirements in academia have resulted in tough competition, causing psychological issues and intense mental pressure on students (3). A study conducted in five provinces in mainland China highlighted that mental disorders in children and adolescents attending school weighted 17.5% (4). Human wellbeing and adequate performance depend upon mental health, and the connection between mental and physical health cannot be separated (5). As per Cardwell (6), the performance-related challenges raised due to severe academic competition are causing depression, anxiety (7), burnout (8), and excessive use of alcohol (9) in students. This decline in students' mental health also affects the quality of their studies and causes poor academic performance (10). According to Thornicroft (11), one can understand the term stigma as a problem of knowledge (ignorance), attitudes (prejudice), and behavior (discrimination). According to Stuart (12), stigma is a global phenomenon, but culture plays a significant role, and certain types of discrimination and different kinds of stigma experiences are influenced by the local culture of a society or institution. When this stigma is coupled with any other mental disorder, it hinders the people facing mental illness's challenges in seeking help, treatment, and care from others (13, 14).

Students who are depressed or experience the symptoms of depression may become trapped in a loop of a negative spiral, and this depression interferes with their studies and results in poor academic performance (15). In a recent study on undergraduate medical science students conducted by Ngasa (16), it was highlighted that depression has no correlation with the grade points average (GPA) of the students, and even when students are depressed, they perform well and get good grades. Anxiety and performance in academia have a nuanced relationship. A study by Wilson (17) revealed that as students' stress, anxiety, and depression level increases, the thoughts of being helpless increase, and thus, the chances of poor academic performance also increases. There are few scholars with contradictory views; Lama 2011 (18) conducted a study and found that the prevalence of anxiety in students acts as a driving force and results in better performance of the students. Another longitudinal study also stressed a similar finding that students with anxiety get good grades (19). A recent study by Dekker et al. focused on optimizing students' mental health and enhancing their academic achievement through the intervention of chatbots, which facilitated the students in prioritizing their academic tasks (20). Shameran (21) explained and identified that mental health disorder causes the students' academic performance to decline. However, the study on students suffering from Attention-Deficit Hyperactivity Disorder concluded that teachers' evaluation of such students is poor, and they acknowledged that these students are unlikely to perform and get good grades (22). Bullying has also been associated with causing depression, anxiety, and a sense of discrimination among students. Research in this area has concluded that bullying causes anxiety and results in poor psychological health, severely affecting a student's overall grade point average (GPA) (23). Researchers have been using the sample of college students, medical students, and the general public and have concluded that individuals in depressed states were reluctant to seek help to overcome their depression and continued to perform lower (24). Furthermore, according to Kerry (25), medical students regarded mental health stigma as a barrier and considered it a sign of their weakness, indicating their inability to perform specific tasks.

In the last two decades, the dynamic growth, progress in information and communication technology, widespread internet accessibility, and the flexibility that online courses offer have made online education an integral part of higher education (26, 27). The literature on active student-centered teaching methodologies highlights numerous strategies, which include flipped classroom (FC) (28), computer-supported collaborative learning (29), problem-based learning (30), and Massive Open Online Courses (MOOCs) (31). Strelan (32), in his research, concluded that FC is the most effective student-centered teaching methodology. According to Meehyun (33), it improves the quality of teaching as well as the quality of learning. Moreover, it enables a learning environment by providing more flexibility (34), improves student engagement (35), increases interaction between student and teacher (36), and increases the academic performance of the student (37). According to Bond (38), FC is gaining rapid popularity in the education sector and has multiple types, such as out-of-class activities focusing on individual learning like field trips and in-class activities of group learning like project-based learning (39). Flipped learning readiness (FLR) is a term that explains the student's readiness and when the students intend to prepare themselves before the class (40) sufficiently. The success or failure of the FC teaching model depends upon the level of FLR, and the low level of FLR can cause the FC model to lose its advantages. The level of students' readiness is crucial to achieving better learning outcomes and understanding the effectiveness of FC-based teaching methods (41, 42).

The literature on flipped learning readiness provides various associations between FLR and performance (43). Numerous studies have presented evidence that students who prefer and are attuned to teacher-centered teaching processes showed low FLR (41, 44, 45). These studies also recommended that students with low FLR might be suffering from mental health issues and thus might need special counseling to escape it and improve their learning skills in before-class activities (46). Moreover, flipped learning is a significant element of a student's academic life (40), and recent studies have also attempted to highlight the significant relationship between FLR and academic achievement (43, 45). However, there is still a dearth of literature testing this relationship among Chinese-English bilingual students in mainland China. Therefore, this research will address the role flipped learning readiness plays among Chinese-English bilingual students in China and their academic performance.

Moreover, the capability of an individual to be expressive, self-regulating, and understand emotions is called emotional competence (47). EC has also been found to be a significant contributing element in academic performance (48–50), and effective functioning in adulthood (51, 52) is developed through socialization during adolescence (53). Due to COVID-19 outbreak, people have suffered inevitable isolation during the lockdown. The adolescents have depicted less awareness, acceptability, and control over their emotions (53, 54) and have faced a tough time emotionally regulating themselves (54, 55). Previous studies on the direct consequences of COVID-19 found an increase in low emotional competence-related mental health issues in adolescents and young adults (56–58).

Furthermore, teenagers must be emotionally competent to deal with the additional emotional pain produced by COVID-19, such as sickness, loss of family, and financial issues during the pandemic (58–60), as well as feelings of anxiety, depression, and sadness (61). Students with solid emotional competence will be able to control their loss, sadness, and stress better in dealing with the new online learning environment (62, 63). Moreover, emotional competence plays different roles in adolescents' and young adults' lives (51, 64), but few studies have differentiated the roles that emotional competence plays in academic performance among Chinese-English bilingual students in mainland China. Therefore, this research will address the role that emotional competence plays among Chinese-English bilingual students in China and their academic performance.

Above mentioned and numerous previous studies have explained the phenomena of depression, anxiety, and burnout resulting in poor performance. However, to our knowledge, no other studies related to mental health stigma (MHS) and students' academic performance have included the cognitive element of flipped learning readiness and emotional competence. Furthermore, little is known about whether students are emotionally competent and ready to learn effectively through flipped learning. Therefore, this study is aimed to see the mediator roles of flipped learning readiness and emotional competence in the relationship between mental health stigma and academic performance.

## Literature review and hypotheses development

### Dual-process theory

Human brains contain numerous interacting cognitive mechanisms that initially came into existence by some analysis in the past (65, 66). However, many individuals are well-known for their logical reasoning, but they are observed to act blindly with time. The theory of the Dual-process says that the reason for the above-mentioned social behavior is a result of two cognitive plans: one that is frequent, although frail, procedures that may operate with no effort, and the others are aimed at efforts that can be slow but lead to accuracy. There can be contradictions in dual-process theories in understanding the true significance of two cognitive systems. In order to understand this, we can take the example of Evans and Stanovich (67) study on dual-process theory. They suggest that all processes came out of multiple cognitive and dual systems, which led to understanding two cognitive mechanisms.

With the improvements in the literature on reasoning, a wide range of psychological processes involving the capability of judging and making decisions have been using dual-process theories (68). research on supervised human learning has also shared similar findings. According to behavioral and neurological research, the human brain has two independent choice systems: a quick, reflexive system based on habits and a slow, deliberate system focused on objectives (69). These systems' processes have been translated into model-based vs. model-free reinforcement learning techniques. A differentiation between model-free and model-based systems has also been proposed to cater to the nature of the two systems proposed to underpin moral reasoning (70). The fear of being stigmatized often governs many facets of human conduct, disgrace, and sanction socially. In many instances, the fear of stigma leads people to hide particular behaviors or activities rather than change their behavior. Hence, we can conclude the study that mental health stigma is taken as a dual cognitive mechanism, and it encourages frequent but misguided cognitive processes.

### Mental health stigma and Chinese-English bilingual students' academic performance

Mental health stigma usually connects to stereotypes regarding mental sickness, which affects our views about ourselves and others. Researchers connect it with postponed treatment, lowering the severity of illness, and suicidality stigma can manifest at several levels (71). For example, social stigma occurs due to labeling and stereotyping, while self-stigma marks internal negative biases that affect an individual's behavior. The stigma considered influential in social activities and behavior is known to be the structure of institutional stigma (72). Mental disorder is usually the result of stigmatization by society, intolerance, and discriminatory behavior (73).

There are immediate effects of mental disorder on an individual and are of multiple types such as functional impairment and under-performance in their daily activities, and if persisted for a long time, it may cause severe physical and mental issues (74, 75). Mental illness could be converted into escalated harmful endings in terms of social health and wealth, like poor living standards, lack of efficiency in work life, and degraded health condition. Impaired academic performance is a direct outcome of both poor mental health and negative experiences in one's life (76), and few critics came up with the idea that mental health programs can lead scholars to attain a high academic performance (77, 78). There are many obstacles to applying this, which involve the requirement of an expert, the enhanced demand for treatments needed to improve mental health, and the stigma and inequality which still can be experienced (77, 79). Therefore, institutions need to employ and embed easy and cheap methods in their systems to enhance students' academic performance and overall wellbeing.

Higher education institutions must participate rigorously to address the issue of mental health because an increasing number of young students with mental health issues are enrolling in the universities, which adds to the pressure universities have to take for their counseling. It is observed in many cases that decreased academic grades result from stressful mental health and lifestyle of the individual (80, 81). It is often seen in universities that mental health issues worsen- 73% of scholars that experienced mental health issues in the past suffered from mental health crises, which lowers the possibility of acquiring a graduation degree. It is observed from the literature that Black and Asian scholars are experiencing more mental health stigma than others worldwide (53). Hence, the following hypothesis is advised to form the above discussion.

H1. Mental health stigma negatively affects Chinese-English bilingual students' academic performance.

### Mental health stigma and flipped learning readiness

The term flipped learning readiness (FLR) refers to the scholars' ability to study and intention to arrange themselves properly (40), and hence it emphasizes the feature of pre-class learning. The decline in scholar FLR is one of the significant concerns that temper the benefits of the FC model. The scholars' readiness should be considered better to understand the value of FC learning (41, 42). Research has shown that scholars do not show signs of better FLR, particularly in teacher-centered processes (37, 41, 44, 45). The overview of past studies enforces that such scholars require well-organized supervision and continuous advice, mainly to enhance the art of learning, which plays a vital role in pre-class activities (46).

Mental health can be taken as one of the alterable factors which should be considered as it is one of the most challenging phenomena occurring in online learning programs (82). The terminology of mental health is considered closely related to the level of distress and disability (83), which can bring personal worries regarding social interactional episodes, like public gatherings and communication with others (84). The study carried out by Keskin (82) highlighted that interaction, collaborations, and interaction with classmates and teachers in flipped classroom strategies result in more, in comparison to face-to-face learning techniques, anxiety and stress among the students. The teachers responsible for online learning sessions are recommended to take advantage of the research and implement FC in an e-learning atmosphere.

Mental health stigma in online learning sessions is related to a fear of negative assessment while exchanging a few words with others (85). The past study observed that the students who intentionally avoid social gatherings fear having a false assessment by others, and as a result, they resist getting engaged in any social activity (86). In the same way, scholars having negative opinions in flipped learning sessions put them in a situation where they fear interacting in social gatherings (85, 87). The students should be engaged in activities that include social communication; for those reasons, negative assessment is considered an essential aspect of online FCs. Most notably, it is emphasized that social nervousness can have a damaging impact while making relationships with others, communicating socially, and performing lectures (82, 88, 89). We may say that scholars neglect to communicate, and having negative assessments may harm social meetings. Unluckily, social anxiety may result in sadness, escaping behavior, and, most importantly, stressfulness when the individual is required to communicate with others in gatherings (89, 90). This problem is related to somatic symptoms, which negatively impact communication. The present study suggests the following hypothesis:

H2. Mental health stigma negatively affects Chinese-English bilingual students' flipped learning readiness.

H3. Flipped learning readiness mediates the relationship between mental health stigma in Chinese-English bilingual students and their academic performance.

### Flipped learning readiness and academic performance

Students' FLR level is vital in uplifting their learning skills (91–93). The past studies explain that the most crucial facet of the FC model is to get prepared for the class before lectures since it plays a vital role in helping the students to focus and increase their concentration on learning (94), to improve their content understanding (37), and improvement of self-learning skills (95). The student's learning skills developed positively through pre-class activities (96, 97). The e-learning FC model involves various learning techniques, written material, and a variety of data which helps improve the hours spent attending the class. Thus encouraging the students to get more involved in the learning process and benefit from the available data and the advanced learning methodologies (93).

Furthermore, the FC model needs scholars to build the ability to manage time efficiently (98). The available research helps to identify the relationship between FLR, sub-branches of FLR, and accomplishment. The present study addresses the following hypotheses:

H4. Flipped learning readiness positively affects Chinese-English bilingual students' academic performance.

### Mental health stigma and emotional competence

Mental illness causes destruction in behavior and emotional and mental performance, which could slow down the capacity of an individual to execute specific responsibilities which he could perform without hesitation and have adverse effects on more than one area of life (99). Stigma is a complicated mechanism that is determined by psychological and sociological aspects. It also involves personality gaps, social parts, and traditional arrangements (100, 101). Contrary to this, Emotional competence/emotional skills/emotional intelligence was projected to explain the central theme. Those with enhanced EI are believed to recognize the emotions of themselves as well as others, elaborate on them in public in a satisfactory way, appreciate the reason and after effects, manage them if they are not suitable for the required aims, and utilize them to improve judgments and events (102). Individuals are going through mental health stigma and struggle to acquire help from others (103, 104). In academic institutes, the acknowledgment of work accommodations depends on a scholar's willingness to acquire the supervisors to provide a helping hand, but this will unveil the mental disorder (105, 106). The stigma relevant to mental illness prevents the suffering person from asking for help and opening up about their mental health status because they fear public stigma (i.e., mental illness-connected discrimination and inequality from society) (105, 107). When a worker discloses a mental disease, he or she may face stigma-related negative behaviors from instructors and students, such as social rejection, prejudice, discrimination, and harassment (104, 108, 109). Adding a few more, the person facing mental health stigma is not capable of identifying and understanding how they feel. As a result, they face severe issues in handling their emotions appropriately, which may worsen their ability to control their stress level and hence develop into further incapability to stay away from such situations (62).

Furthermore, a study show connection between emotional intelligence, anxiety, and depression (110), and a researcher found that after 1 year, exhaustion is the only reason for self-appraisal emotion relevant to the EI aspect (111). The discussed data unveils that psychological health has a crucial role in many aspects of life and is critically related to EI. Hence, the present study develops the following hypothesis:

H5. Mental health stigma negatively affects Chinese-English bilingual students' emotional competence.

### Emotional competence and academic performance

Emotional competency is conceptualized from the ability model Mayer gave (47) and is defined as a mental aptitude for sensing, accepting, utilizing, and operating self-emotions and other individuals' emotions. The past study relates that emotionally intelligent individuals are observed to possess good psychological behavior management (e.g., more happiness and self-motivated as well as low levels of stress in their life (112, 113) as well as a flourishing life with elevated satisfaction level (112, 114–116). Former researchers resulted that from an educational point of view, the improvements in emotional competencies could be utilized as a helpful tool for elevating the psychological modifications and social relationships in society (117–119). However, facts show that emotional intelligence is reasonably responsible for the academic achievements of scholars (114, 120). Others suggested that individuals having better emotional intelligence could manage the academic burden more efficiently (e.g., anxiety about the examination and frustration about the future), and these capabilities are found to be more helpful when communicating amongst supervisors and teammates (120). Hence, a few years back, research unveiled many hidden techniques, like managing emotions or self-directed learning (118, 120, 121), that could relate to academic performance and emotional intelligence.

Furthermore, MacCann (120) has declared the fact that a few non-cognitive traits like emotional intelligence might affect the academic performance of the individual because of alteration in education (e.g., teamwork activities have increased to a certain extent), the need to understand conflict management skills between group-mates, decision making power, or solution finding in a team. On the other hand, the relationship between emotional intelligence and academic performance still needs to be explored. In the present study, we investigated the possible mediating role of flourishing and the moderating role of teacher-student relationships in the relevance of emotional intelligence and academic performance.

H6. The emotional competence of Chinese-English bilingual students positively affects their academic performance.

H7. Emotional competence positively mediates the relationship between mental health stigma in Chinese-English bilingual students and their academic performance.

**Figure 1:**
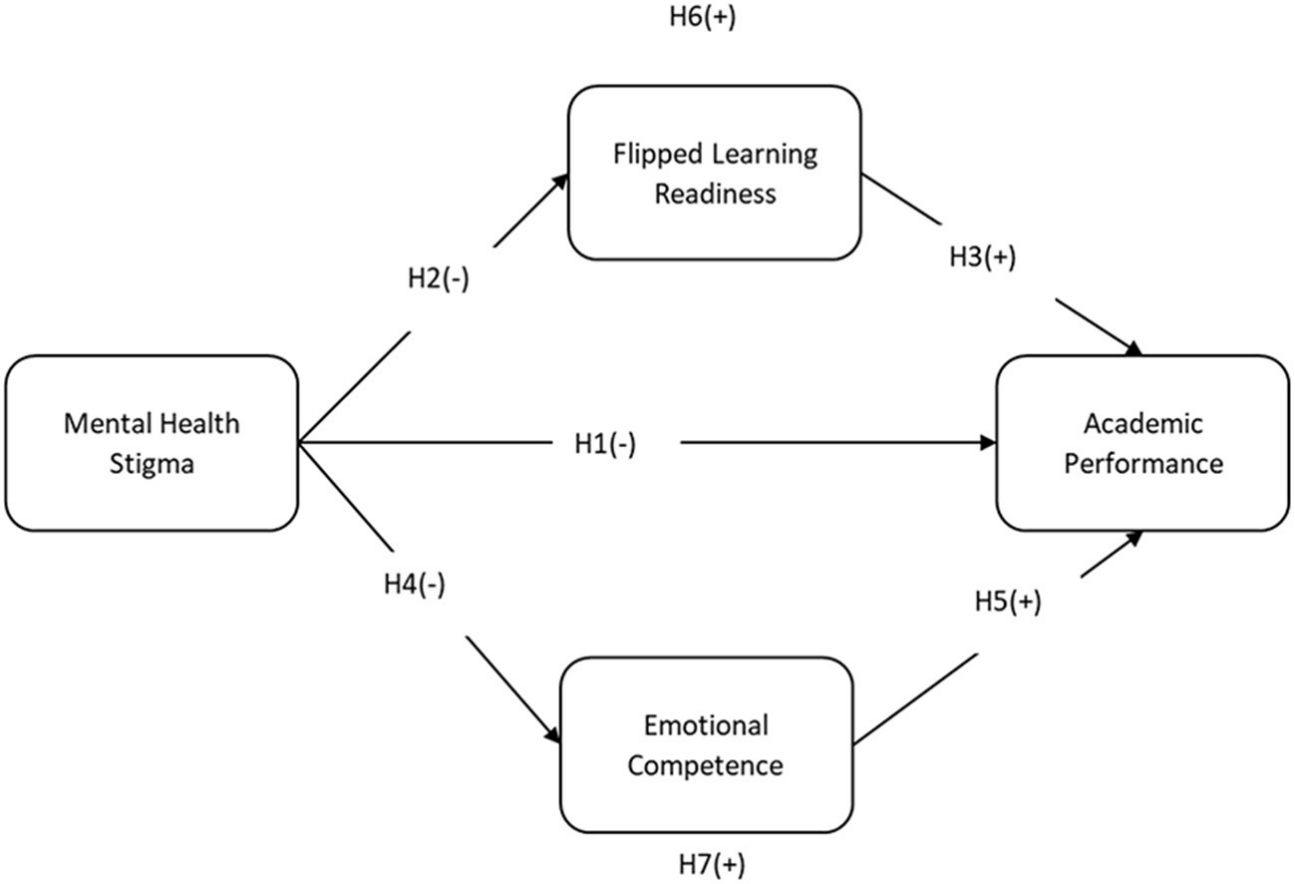
Theoretical framework. (+) = positive relationship; (–) = negative relationship; H1, H2, H3, H4, H5 = direct relationships hypothesis; H6, H7 = mediating role relationships.

In Figure 1, the (+) sign indicates the positive relationship between the variables, the (–) sign indicates the negative relationship, and the Hypothesis number (i.e., H6 and H7) mentioned above, the rectangular shape of the variable, indicates the mediators.

## Methodology

The research was handled methodologically with descriptive and relational reasons. The information was gathered from universities in five provinces of mainland China, namely Zhejiang, Hubei, Jiangxi, Shanghai, and Guangdong. The data was collected using an online survey approach and a survey questionnaire. Prior to data collection, respondents were educated. There were two sections to the questionnaire. The respondent's demographic information, like age, gender, degree program, year of study, and grade point average, were obtained in the first part. In the second portion, we offered questions about each of our variables. The response to each question item was obtained using a five-point Likert scale. The non-probability purposive sampling approach was used to acquire data. The questionnaire was e-mailed to 1,080 Chinese students studying English as a major. Only 505 respondents filled out the questionnaire (47% response rate); valid information was gathered only from 448 respondents. SPSS was used for fundamental analysis, while AMOS v.24 was used for structural equation modeling (SEM) to test the hypothesis.

### Questionnaire design

The questionnaire in this study was designed on a five-point Likert scale. This form of questionnaire is said to help gather data from a large population in a trustworthy manner. Indeed, a survey-based questionnaire is vital for collecting data from respondents since it is simple to disseminate and collect the questionnaire for analysis. As a result, the current study used the same data-gathering strategy. The scale questions were collected from several studies with careful attention and face validity to gather data from respondents. The research used a nine-item measure for mental health stigma (122).

Further, the twenty-six scale items to measure the flipped learning readiness were used. The scale was developed by Hao (41) and later adapted to the Turkish context by Yildiz-Durak (123) to measure university students' readiness level for flipped learning. In the same way, the twenty-one scale items for emotional competence were adopted from the study (124). Understanding that mental health stigma plays a critical role in people's mental development and performance in day-to-day activities is critical. Also, these scale items were previously used by different studies to understand the role of mental health education in the better academic achievement of the students in class activities.

Similarly, the scale questions for students' mental health stigma were carefully chosen to recognize the importance of students' psychological aspects. To adequately quantify the link, this study used scale items that were carefully chosen. Furthermore, the flipped learning readiness scale items were used to assess the mediating function of flipped learning readiness in the link between mental health stigma and academic performance in Chinese-English bilingual students. Finally, the scale items for emotional competence were used to collect data to understand its relationship to Chinese-English bilingual students' academic performance and to check its mediating role between mental health stigma and academic performance. Furthermore, the experts' opinions were considered when testing the face validity of these scale items, and the research experts were contacted to provide their opinions on the face validity of the questionnaire. As a result of receiving positive responses from various experts, these scale items were incorporated into the questionnaire to collect data from the target respondents effectively.

### Data collection process

In this section of the study, the detail of the data collection process is presented. Firstly, the current study respondents were Chinese national students at different universities in mainland China, with Chinese as their mother language and studying English as a major. Therefore, the non-probability purposive sampling technique was adopted, and students enrolled in English as a major degree were e-mailed the questionnaire. Notably, the respondents' consent was taken to respond to the questionnaire. Further, with the positive response from the individual students taking English as a major, the questionnaire was distributed to them, and they were provided with a brief introduction to the study to get familiarity with it. Also, the individuals were allowed to ask any question related to the study, with difficulty responding to the questionnaire. This way, the response rate for mental health studies was 47%. In this regard, 1,080 questionnaires were e-mailed to the students. Finally, 505 questionnaires were received from the students, and only 448 were fit to be analyzed for this study. Finally, the students were thanked for their precious time, participation, and contribution to the study.

## Findings

### Respondents' demographic

This study researched students learning English at different universities in China, so all the respondents were students studying English as a major. Table 1 shows the demographic characteristics of the respondents, where 47.6 percent of the students were male, whereas 52.4 percent were female. Among the respondent, 61.3 percent belonged to the 18 to 21 years age group, 16.3 percent were from the 21–24 age group, and 14.4% were from 24–27 years of age. In total 71% of these respondents were undergraduate students, and 29% mentioned postgraduate students. Out of our total of 448 respondents, 16.5% mentioned studying English for 1–3 years, whereas the vast majority of 71.5% were studying for 4–6 years. We also enquired about any other student in their knowledge who has been going through any mental health stigma, and 27.2% said “yes” they know a student who is going through a mental health stigma/illness.

**Table 1:** Respondents' demographic characteristics.

**Variable**	**Categories**	**Frequency**	**Percentage**
Gender			
	Male	235	52.4
	Female	213	47.5
Age			
	18–21 years old	275	61.3
	21–24 years old	73	16.3
	24–27 years old	65	14.4
	27–30 years old	21	4.7
	>30-year-old	14	3.3
Civil Status			
	Single	384	3.7
	Married	47	3.9
	Separated/Divorced	17	58.5
	Widow/Widower	0	27.3
Year of Study			
	Freshman	72	16
	Sophomore	183	41
	Juniors	121	27
	Seniors	72	16
Degree Program			
	Bachelor's degree	318	71
	Master's degree	116	26
	Doctoral degree	14	3
How long have you been speaking English?		
	1–3 years	74	16.5
	4–6 years	320	71.5
	Others	54	12
Know someone with mental health			
	Yes	122	27.2
	No	326	72.8
Has or had a mental illness			
	Yes	128	28.5
	No	320	71.5

### Reliability, validity, and measurement model tests

The convergent validity of the constructs in the present study was addressed based on the criteria suggested by Hair et al. (125). These criteria are: (1) the factor loadings for the measurement items of all the constructs must exceed 0.60, (2) the value of composite reliability for each of the constructs must be equal to or larger than 0.70, and (3) the value of the average variance extracted (AVE) for each of the constructs is more extensive than 0.50. An item from the resistance construct was removed due to its low loading (i.e., less than 0.60). After removing such items, the outputs in Table 2 have shown that the measurement model fulfilled the requirements of convergent validity. Therefore, convergent validity has been conclusively established for this study.

**Table 2:** Convergent validity, reliability and factor loadings.

		**Items**	**Scale items**	**Loading**	**AVE**	**CR**	**α**
Flipped learning readiness	Student control and self-directed learning	I can manage my own learning process.	SCSDL1	0.735	0.859	0.799	0.879
		I set my own learning goals.	SCSDL2	0.728			
		I repeat teaching materials according to my learning needs.	SCSDL3	0.846			
		I have higher expectations for my learning performance.	SCSDL4	0.707			
		I prepare my own study plan and put it into practice.	SCSDL5	0.771			
		While doing the preliminary study, I do not get distracted by other activities in the learning environment (instant chat, surfing on social networks, games on the Internet, etc.).	SCSDL6	0.755			
		I ask for help when I encounter problems in the learning environment.	SCSDL7	0.786			
		I have self-discipline.	SCSDL8	0.692			
	Technology self-efficacy						
		I can download files from the Internet.	TSE1	0.665			
		I can use media players (Media Player) to listen or watch online multimedia materials.	TSE2	0.811			
		I can use document viewing software (Adobe Reader etc.) to view learning materials.	TSE3	0.735			
		I can use online note-taking technologies (Colornote) to take notes or access their notes	TSE4	0.749			
		I can use e-mail to communicate.	TSE5	0.72			
		I can use instant messaging software (like Skype, WhatsApp) to communicate with people.	TSE6	0.751			
		I can use information technologies to organize the learning materials I seek online	TSE7	0.831			
		I can identify what I need from the information in online resources.	TSE8	0.834			
		I can determine the accuracy and reliability of online information.	TSE9	0.779			
	In-class communication self-efficacy						
		I feel confident when asking questions in lessons.	ICCSE1	0.754			
		I feel confident while expressing myself in lessons.	ICCSE2	0.824			
		I feel confident in discussions with my teacher about the subject in lessons.	ICCSE3	0.835			
		I feel confident in discussions with my friends about the subject in the lessons.	ICCSE4	0.748			
	Motivation for learning						
		In the learning environment, my mistakes allow me to learn new things.	ML1	0.813			
		I like to share my ideas with others in a learning environment.	ML2	0.82			
		I have motivation to learn in the learning environment.	ML3	0.71			
	Pre-study						
		I am ready to do a preliminary study by listening to the lessons recorded by my teacher.	PS1	0.809			
		I am ready to do a preliminary study by watching online videos on the subject.	PS2	0.838			
	Perceived Mental Health Stigma						
		Most people believe that people with depression could snap out of it if they wanted.	PMHS1	0.849	0.933	0.949	0.789
		Most people believe that depression is a sign of personal weakness.	PMHS2	0.873			
		Most people believe that depression is not a real medical illness.	PMHS3	0.821			
		Most people believe that people with depression are dangerous.	PMHS4	0.865			
		Most people believe that it is best to avoid people with depression so you don't become depressed yourself.	PMHS5	0.867			
		Most people believe that people with depression are unpredictable.	PMHS6	0.911			
		If they had depression, most people would not tell anyone.	PMHS7	0.871			
		Most people would not employ someone they knew had been depressed.	PMHS8	0.915			
		Most people would not elect for an individual if they knew had been depressed.	PMHS9	0.878			
Emotional competence	Identification-Self	When I am touched by something, I immediately know what I feel	IntraEC1	0.847	0.712	0.794	0.791
	Identification-Self	When I feel good, I can easily tell whether it is due to being proud of myself, happy or relaxed.	IntraEC2	0.876			
	Understanding-Self	I do not always understand why I respond in the way I do (R)	IntraEC3	0.835			
	Understanding-Self	When I am feeling low, I easily make a link between my feelings and a situation that affected me.	IntraEC4	0.86			
	Expression-Self	I find it difficult to explain my feelings to others even if I want to ®	IntraEC5	0.847			
	Expression-Self	I am good at describing my feelings	IntraEC6	0.881			
	Regulation-Self	When I am angry, I find it easy to calm myself down	IntraEC7	0.836			
	Regulation-Self	I find it difficult to handle my emotions (R)	IntraEC8	0.898			
	Use-Self	My emotions inform me about changes I should make in my life	IntraEC9	0.826			
	Use-Self	I never base my personal life choices on my emotions (R)	IntraEC10	0.815			
	Identification-Others	I am good at sensing what others are feeling	InterEC1	0.845			
	Identification-Others	Quite often I am not aware of people's emotional state (R)	InterEC2	0.783			
	Understanding-Others	I do not understand why the people around me respond the way they do (R)	InterEC3	0.804			
	Understanding-Others	Most of the time, I understand why the people feel the way they do	InterEC4	0.885			
	Listening-Others	Other people tend to confide in me about personal issues	InterEC5	0.813			
	Listening-Others	I find it difficult to listen to people who are complaining (R)	InterEC6	0.809			
	Regulation-Others	When I see someone who is stressed or anxious, I can easily calm them down	InterEC7	0.838			
	Regulation-Others	If someone came to me in tears, I would not know what to do (R)	InterEC8	0.762			
	Use-Others	I can easily get what I want from others	InterEC9	0.817			
	Use-Others	If I wanted, I could easily make someone feel uneasy	InterEC10	0.733			
	Academic Performance	GPA	GPA	1.000	1.000	1.000	1.000

Factor loadings, average variance extracted (AVE), composite reliability (CR), and Cronbach's Alpha (α) indicate the convergent validity of measurement model.

**Figure 2:**
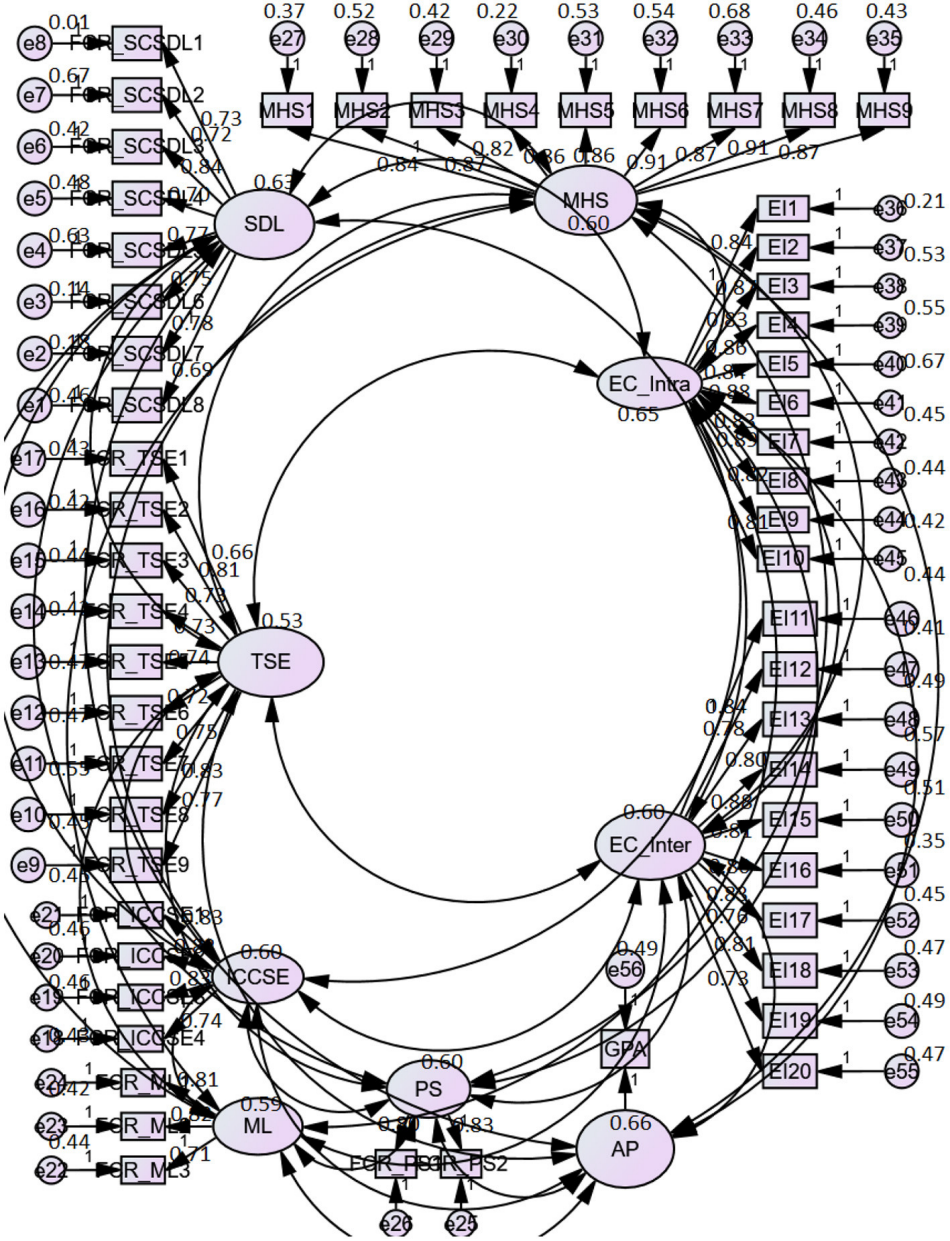
Measurement model.

There was no multicollinearity issue in the data set as the variance inflation factor (VIF) values were between 1.004 and 1.142, and tolerance values ranged between 0.876 and 0.999, as suggested by Hair et al. (126), which is an apparent absence of multicollinearity. In addition, the discriminant validity for the present study was assessed using the guideline proposed by Fornell et al. (127), which is based on comparing the squared root of AVE values against the maximum shared variance (MSV) values of its own and the variance of other constructs. The discriminant validity results highlighted in Table 3 show that the values of the squared root of AVE (diagonal entries in bracket) are smaller than the values of MSV on their own and more remarkable than the variance shared between any two constructs (off-diagonal entries in italic). The measurement model has been graphically presented as Figure 2.

**Table 3:** Discriminant validity.

	**MSV**	**MHS**	**FLR**	**EC**	**AP**
**MHS**	0.202	***0.859***			
**FLR**	0.193	*0.842*	***0.875***		
**EC**	0.02	*0.853*	*0.733*	***0.844***	
**AP**	0.185	*0.837*	*0.757*	*0.782*	***1.000***

The values of the squared root of AVE (diagonal entries in bracket) are smaller than the values of MSV on their own and more remarkable than the variance shared between any two constructs (off-diagonal entries in italic).

### Structural equation modeling

The next step is to check the causal model. We first run the structural equation modeling on our basic model. At first, the model was not fit, and the results were abysmal. Then, to ensure that the data has no poor fit values, we followed the recommendations by Hair et al. (126) and re-estimated the model fitness values. Table 4 shows the model fitness test's initial model and specified values. Furthermore, the results are evident after the modified model is fit for further analysis.

**Table 4:** Measurement model fitness values.

**CFA indicator**	**Threshold value**	**Initial model**	**Modified model**
CMIN/DF	≤3	5.19	1.918
GFI	≥0.80	0.825	0.86
AGFI	≥0.80	0.766	0.842
CFI	≥0.90	0.912	0.96
RMSEA	≤0.08	0.1	0.055
NFI	≥0.90	0.914	0.902
TLI	≥0.90	0.886	0.941
IFI	≥0.90	0.913	0.94
PCLOSE	>0.05	0.000	0.081
SRMR	<0.08	0.091	0.064

Values of Model fitness indices of initial model and modified (re-specified) model.

### Hypothesis testing

**Figure 3:**
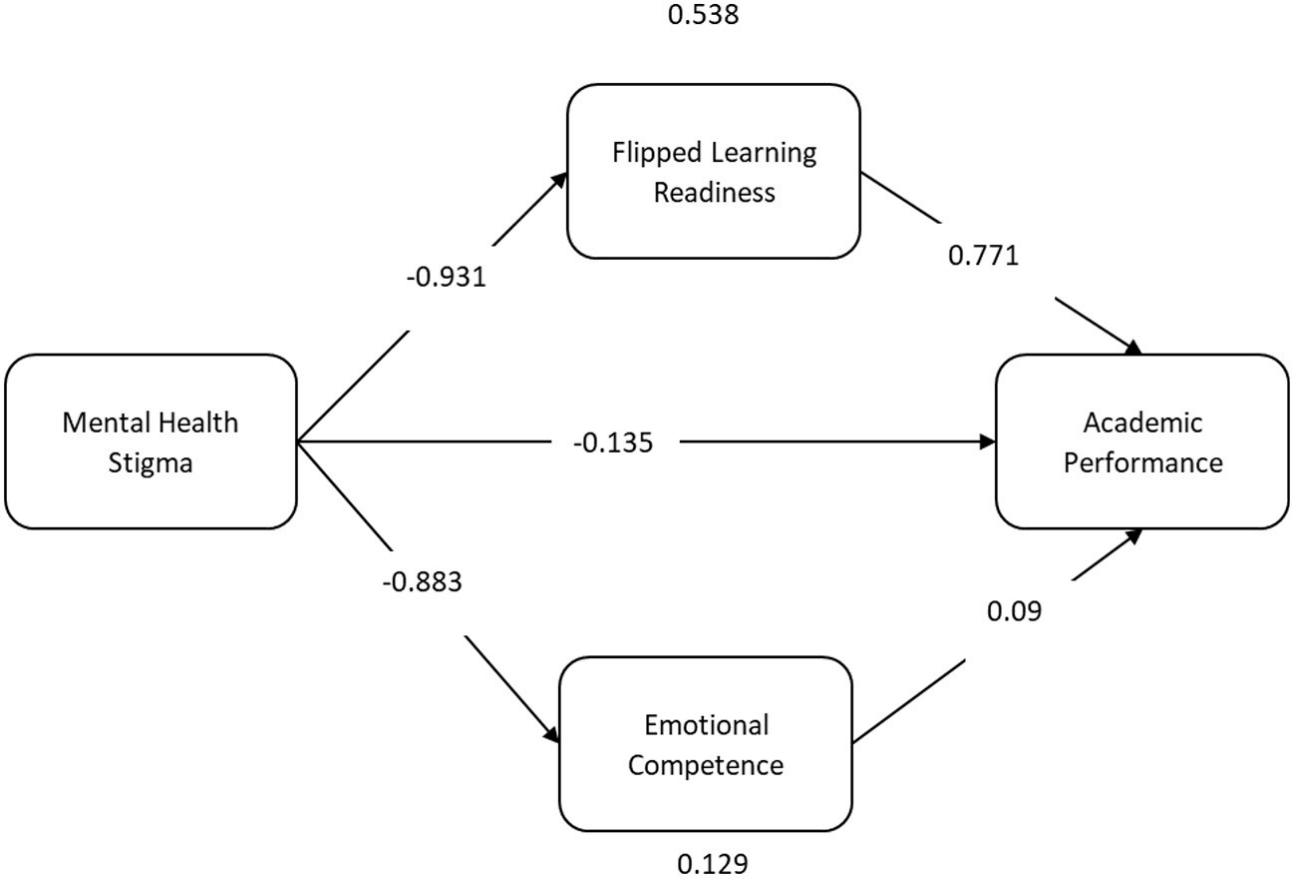
Structural model.

We first checked the direct effects of our proposed hypothesis, as shown in Figure 3.

The first hypothesis is a relationship between mental health stigma and Chinese-English bilingual students' academic performance. Moreover, the estimated values show a negative relationship between mental health stigma and students' academic performance with the values (β = −0.135 and *p* < 0.001). The second hypothesis states that a relationship between mental health stigma and flipped learning readiness and values (β = −0.931 and *p* < 0.001) show a negative relationship. Our proposed third hypothesis was to test the positive relationship between flipped learning readiness and students' academic performance, and values (β = 0.771 and *p* < 0.001) show a positive relationship. H4 was to test the negative relationship between mental health stigma and students' emotional competence, and values (β = −0.883 and *p* < 0.001) show a negative relationship. Our last direct hypothesis, H5, was to test the positive relationship between emotional competence and students' academic performance, and values (β = 0.09 and *p* < 0.001) show a positive relationship. All the direct hypothesis results are shown in Table 5.

**Table 5:** Direct path effect coefficients.

**Hypothesis**	**Structural relationships**	**Coefficient (β)**	**SD**	**t Statistics**	***p-*value**	**Decision**
H1	MHS –> AP	−0.135	0.041	3.233	0.001	Supported
H2	MHS –> FLR	−0.931	0.016	58.265	0.000	Supported
H3	FLR –> AP	0.771	0.036	21.291	0.000	Supported
H4	MHS –> EC	−0.883	0.023	39.12	0.000	Supported
H5	EC –> AP	0.09	0.029	3.019	0.003	Supported

MHS, Mental health stigma; FLR, Flipped learning readiness; EC, Emotional competence; AP, Academic performance.

Furthermore, according to the results shown in Table 6, flipped learning readiness mediates in the relationship between mental health stigma and students' academic performance (β = 0.538, t = 20.85); therefore, H6 is accepted. Also, according to the results, emotional competence mediates in the relationship between mental health stigma and students' academic performance (β = 0.129, t = 3.024). Hence H7 is also accepted.

**Table 6:** Direct and indirect path effect coefficients (mediation results).

**Hypothesis**	**Indirect Path**				**Mediation**	***t*-value**	**Decision**
**Path**	**β**		**Path**	**β**	**effect β**		
H6	MHS→ FLR	−0.931	FLR→ AP	0.771	0.538	20.85	Supported
H7	MHS→ EC	−0.883	EC→ AP	0.09	0.129	3.024	Supported

## Discussion

This study aimed to investigate the association between Chinese-English bilingual students' mental health stigma and academic performance. Our study is a small contribution to academic performance literature (82, 83). Furthermore, the current study also validates the dual-process theory, where people can reason in ways consistent with the prescriptions of logic; they often do not (65, 66). Therefore, the present study put forward evidence that mental health stigma in bilingual students affects their academic performance and puts them in a position to be unwilling to perform in face-to-face classes, whereas when they have the opportunities to interact through flipped learning, their performance is must better. Similarly, when the mental health stigma is mediated by emotional competence, it has a positive relationship with academic performance (128).

The results of H1 indicated that the students believe that depression is a weakness and not an actual medical illness. Still, it is dangerous for themselves and others to avoid someone with any mental disorder and not tell anyone if they are suffering from depression, have developed stereotypes, or show poor academic performance. Their grade point average is low, and they face severe challenges in studying and concentrating (80). The second hypothesis tested the relationship between mental health stigma and flipped learning readiness. The results highlighted that the students who believe that mental disorder is dangerous for themselves and others tend to avoid interaction with others through any means because they believe that if they contact others, who have depression, they might also become depressed. It resulted from poor flipped learning readiness. Such students lacked the confidence to communicate with others. They were unable to prepare their study plans, were not self-disciplined, could not use technology very well for the learning process, and most importantly, had fragile motivation to study through flipped learning. The findings that the mental health stigma negatively affects flipped learning readiness align with the research of Hwang et al. (85), who mentioned that individual fear when it comes to communication and public speaking. Also, Mental health stigma in online classroom environments is associated with the fear of negative evaluation when communicating with others (85).

The results of H3 highlighted that students who have shown control and adopted self-directed learning could manage their learning process and believe they can use technology to learn and improve their academic performance. The results also indicated that such students could determine the accuracy of online study material and have shown communication self-efficacy by positively interacting in the flipped learning environment. They were confident while expressing their thoughts about the lesson and discussing it with their teachers and classmates. Furthermore, they were well prepared for the online recorded lectures before class, and their motivation for learning resulted in a good GPA. Therefore, it mentioned that the students' flipped learning readiness had improved the academic performance of Chinese-English bilingual students (43).

The results of the fourth hypothesis tested the relationship between mental health stigma and emotional competence. The results mention that students who believe that depression is a sign of their weakness do not interact with others very much, and they consider mental disorders an actual disease and think they cannot escape it. This depression makes them believe that those suffering from depression become unpredictable. As a result, students' self-identification, self-expression, self-regulation, and usage of their full potential reduces. Such students feel low, cannot link feelings with a situation to affect it, and can easily make others uneasy. Therefore, the mental health stigma in students reduces their emotional competence. The negative impact of mental health stigma on emotional competence proves the results of previous research (62, 104).

The fifth hypothesis test results suggest a positive relationship between student's emotional competence and academic performance. Based on the results, it is clear that students understand and respond better when they feel good. They are generally good at expressing their ideas and feelings, have self-control over their aggression, and know how to keep calm. They are also good at sensing what others feel and why people around them respond in a particular way. They know how to get things done by others. All of these contribute to their better performance and result in a good GPA. The findings were similar to Maria (110).

The present study also highlighted that flipped learning readiness is a mediator in the relationship between mental health stigma and students' academic performance. The significant direct and indirect effects indicated the mediating role of flipped learning readiness. It opens up windows of opportunities for the academic institutions to ensure the students suffering from any kind of depression or mental health disease are equipped enough to use technology for learning through recorded lectures or online classes and that their students are motivated enough to be a part of these flipped learning strategies, as this will enhance the academic performance of the students (33, 129).

Furthermore, the last hypothesis was to test the mediator role of emotional competence. The significant direct and indirect effects show that emotional competence plays a significant role in mediating mental health stigma and academic performance. Keeping these results in view, the academic institutions devise comprehensive plans for the students to enhance their emotional competence by ensuring that the students who are unwilling to understand and develops stereotypes about mental disorder should be capable of calming themselves under challenging situations and could regulate their emotions, understand others and most importantly listen to others. If these traits are developed, they can have good academic grades (128, 130).

## Implications

### Theoretical implications

This paper makes a significant contribution to the mental health literature and the dual-process theory by considering the dual cognitive processes of with and without efforts (i.e., fast thinking approach and time-consuming thinking approach), which results in both negative and positive outcomes in investigating the mental health stigma among Chinese-English bilingual students. However, the fallible heuristics psychological strategy opted for by the students that are the result of an automatic process initiated based on some specific purpose and see their outcomes in the shape of average grade points students achieve (23). Few studies have been conducted to study the impact of mental health using the dual process model. Thus, the significant contribution of the study is to investigate the mental health stigma and its effects on Chinese-English bilingual students' academic performance using the dual-process theory. The results recommend that the students who have fears and develop stereotypes about themselves or others make such hasty decisions making no effort, and such thoughts undermine their performance. Furthermore, we also tested the mediating role of emotional competence and flipped learning readiness in the relationship between mental health stigma and academic performance. This explains that once the students start making cognitive efforts, their fears are reduced and they start thinking constructively, enhancing their academic performance.

### Practical implications

Current research findings are beneficial for improving bilingual student's academic performance, as the empirical evidence paved the guidelines for practitioners to mentor students with mental health awareness issues and ultimately achieve optimal academic outcomes. The findings suggest that the instructors must devise their lectures using the flipped learning schemes because this facilitates underperforming students by offering additional learning resources. The FL model of teaching students suffering from mental stigmas can receive extra relevant materials, study them before class, and gain confidence to participate in class activities and communicate better with others (43). These flipped learning techniques also provide flexibility to such students to learn at their ease. Classroom activities like team tasks, breaking the ice etc., in flipped learning can encourage such students to participate positively, ultimately enhancing their learning and producing good grades. This study also supports the teachers by highlighting how they can utilize flipped learning activities using multiple teaching approaches through multiple modes of teaching to improve the grade of their students. The study also helps teachers design innovative curricula for their students to increase their self-identification to develop self-regulation by designing and planning classroom activities that motivate students to express themselves and use these to bring positive changes to their lives. Such activities also provide the students the platform to understand others, listen to their views, regulate them, and make others do a particular thing. Researchers might continue to seek measurement-related problems when reviewing the literature on mental health stigma. To overcome mental health stigma, students require mental healthcare mentors who can develop mental health awareness to help students become self-directed and self-regulated to perform academically (25). Therefore, the students are capable of designing self-directed learning plans, can set their own academic goals, are remain self-disciplined. Alternatively, the underperforming students struggling with mental health stigma require institutional support designed to offer mental wellbeing-related training sessions to overcome stereotypes, discrimination, or any other negative attributes that they or the society attach to them.

## Limitations and future directions

There are a few limitations of this study. Primarily, the study has been conducted using the data collected from Chinese university students who are studying English as a significant subject. Therefore, the results could not be generalized as there could be other culture-related, individually related, or psychological factors that can generate different results under different situations. Additionally, the data can be collected from other Chinese who have nationality from English-speaking countries or have studied abroad for a couple of years and then returned to China for work or family. The current study is a cross-sectional study and thus does not correctly explain the causal relationships between proposed variables. Therefore, a longitudinal study is recommended for deep insights into the phenomenon. In this study, we operationalized the mental health stigma as perceived mental health stigma and took a unidimensional scale of it. Previous research suggested that mental health has two dimensions personal stigma and perceived stigma (122). Thus, future research can be conducted these both dimensions. Moreover, flipped learning techniques are the modern teaching methods, and it is not easy for the students to use used easily and quickly. Therefore, it is vital to observe what role institutional support plays (131) in developing such skills in students that influence their learning outcomes.

## Conclusion

This study aimed to explore the relationship between mental health stigma and academic performance of the students studying in Chinese universities who are enrolled in English as a significant subject known as the Chinese-English bilingual. The study also explained the phenomenon of flipped learning readiness and emotional competence mediating the mental health stigma and students' academic performance. Thus, this study contributes to the literature on the academic performance of Chinese-English bilingual students. Our empirical results highlighted that students suffering from anxiety, depression, inability to understand things, or any other mental illness/disorder were performing poorly, and their academic performance was suffering due to these social or public stigmas (4). It highlights the importance that institutions must take initiatives to ensure that students with any mental health stigma should get proper counseling and guidance to improve their academic performance. The results also indicated that students who are good at technology (like skype, media players, adobe reader, WhatsApp etc.) are motivated to learn through modern ways of teaching, and their readiness is validated through improved academic performance (43). The study also revealed that the students who identify, regulate, express, and use self and others have high emotional competence, and such students have performed very well in their classes and shown good overall academic performance as well (20). The current study empirically tested flipped learning readiness and emotional competence as mediators. Moreover, the significant results create opportunities for academic institutions to build readiness and emotional competence in the students to improve their academic performance in terms of grade points average.

## Data availability statement

The raw data supporting the conclusions of this article will be made available by the authors, without undue reservation.

## Author contributions

The author confirms being the sole contributor of this work and has approved it for publication.

## Conflict of interest

The author declares that the research was conducted in the absence of any commercial or financial relationships that could be construed as a potential conflict of interest.

## Publisher's note

All claims expressed in this article are solely those of the authors and do not necessarily represent those of their affiliated organizations, or those of the publisher, the editors and the reviewers. Any product that may be evaluated in this article, or claim that may be made by its manufacturer, is not guaranteed or endorsed by the publisher.
